# 4-Hydr­oxy-3-nitro­benzaldehyde

**DOI:** 10.1107/S1600536808011148

**Published:** 2008-04-26

**Authors:** Mohd. Razali Rizal, Isha Azizul, Seik Weng Ng

**Affiliations:** a Department of Chemistry, University of Malaya, 50603 Kuala Lumpur, Malaysia

## Abstract

The hydroxyl group in each of the two independent mol­ecules of the title compound, C_7_H_5_NO_4_, participates in two O—H⋯O hydrogen bonds, *viz.* one intra­molecular bond to the nitro group and one inter­molecular bond to the aldehyde group of the same mol­ecule in the next unit, resulting in a linear chain structure. The dihedral angle between the aromatic ring and the nitro group is 10.9 (3)° in one mol­ecule and 9.9 (2)° in the other.

## Related literature

For the structure of 2-nitro­phenol, see: Iwasaki & Kawano (1978 ▶). For the structure of 4-hydroxy­benzaldehyde, see: Jasinski *et al.* (2008 ▶).
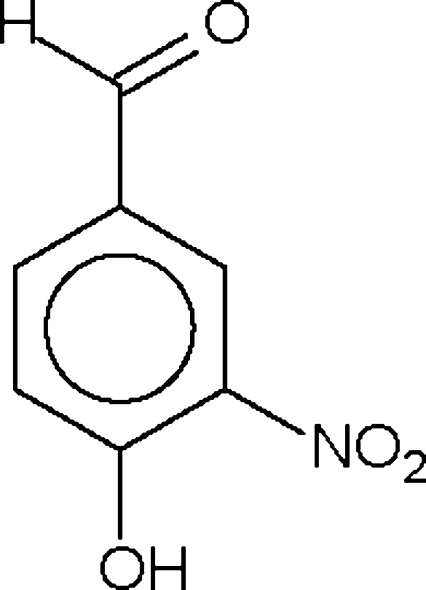


## Experimental

### 

#### Crystal data

C_7_H_5_NO_4_
*M* *_r_* = 167.12
Triclinic, 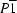

*a* = 8.042 (1) Å
*b* = 8.036 (1) Å
*c* = 12.242 (2) Å
α = 71.975 (2)°
β = 70.820 (2)°
γ = 67.323 (2)°
*V* = 674.1 (2) Å^3^
*Z* = 4
Mo *K*α radiation
μ = 0.14 mm^−1^
*T* = 100 (2) K
0.40 × 0.05 × 0.05 mm

#### Data collection

Bruker SMART APEX diffractometer
Absorption correction: none
4245 measured reflections
3068 independent reflections
2134 reflections with *I* > 2σ(*I*)
*R*_int_ = 0.016

#### Refinement

*R*[*F* ^2^ > 2σ(*F* ^2^)] = 0.045
*wR*(*F* ^2^) = 0.121
*S* = 0.99
3068 reflections
225 parameters
2 restraints
H atoms treated by a mixture of independent and constrained refinement
Δρ_max_ = 0.31 e Å^−3^
Δρ_min_ = −0.32 e Å^−3^

### 

Data collection: *APEX2* (Bruker, 2007 ▶); cell refinement: *SAINT* (Bruker, 2007 ▶); data reduction: *SAINT*; program(s) used to solve structure: *SHELXS97* (Sheldrick, 2008 ▶); program(s) used to refine structure: *SHELXL97* (Sheldrick, 2008 ▶); molecular graphics: *X-SEED* (Barbour, 2001 ▶); software used to prepare material for publication: *publCIF* (Westrip, 2008 ▶).

## Supplementary Material

Crystal structure: contains datablocks global, I. DOI: 10.1107/S1600536808011148/fl2191sup1.cif

Structure factors: contains datablocks I. DOI: 10.1107/S1600536808011148/fl2191Isup2.hkl

Additional supplementary materials:  crystallographic information; 3D view; checkCIF report

## Figures and Tables

**Table 1:** Hydrogen-bond geometry (Å, °)

*D*—H⋯*A*	*D*—H	H⋯*A*	*D*⋯*A*	*D*—H⋯*A*
O4—H4*o*⋯O1^i^	0.84 (1)	2.13 (3)	2.676 (2)	122 (3)
O4—H4*o*⋯O3	0.84 (1)	1.91 (2)	2.638 (2)	144 (3)
O8—H8*o*⋯O5^ii^	0.84 (1)	2.10 (3)	2.687 (2)	128 (3)
O8—H8*o*⋯O7	0.84 (1)	1.94 (2)	2.635 (2)	139 (3)

Symmetry codes: (i) 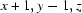; (ii) 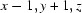.

**Fig. 1.**
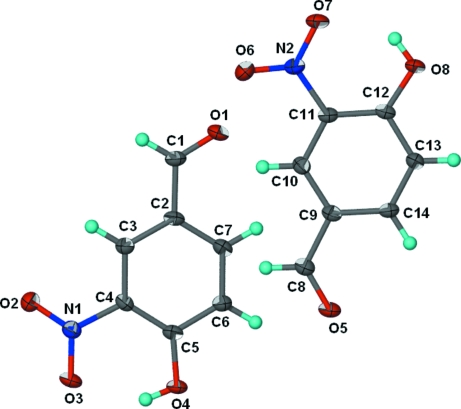
Thermal ellipsoid plot of the two independent molecules of the title compound at the 70% probability level; hydrogen atoms are drawn as spheres of arbitrary radius.

**Fig. 2.**
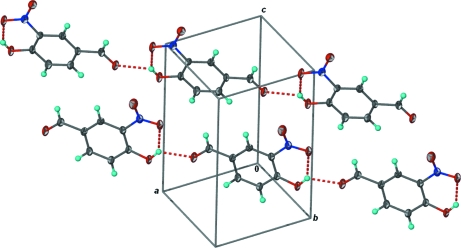
Chain structure of 4-hydroxy-3-nitrobenzaldehyde. Dashed lines denote hydrogen bonds.

## supplementary crystallographic information

### Comment

A nitro group either *ortho* or *para* to a hydroxyl group can significantly increase the acidity of the resulting phenol. The crystal structures of a large number of 2-nitrophenol compounds have reported; the title compound represents another example. The hydroxyl group of the two independent molecules of the title compound (Fig. 1) is intramolecularly linked to the nitro group by an O–H···O hydrogen bond; the hydroxy group is intermolecularly linked to the aldehyde group of the molecule in the next unit cell by an similar hydrogen bond to result in a linear chain structure (Fig. 2). 2-Nitrophenol itself features an intramolecular hydrogen bond of 2.602 Å (Iwasaki & Kawano, 1978). On the other hand, 4-hydroxybenzaldehyde exists as a hydrogen-bonded chain [O–H···O 2.731 (2) Å] (Jasinski *et al.*, 2008).

### Experimental

The commercially available compound (Sigma Aldrich) was recrystallized from water.

### Refinement

Carbon-bound H-atoms were placed in calculated positions (C—H 0.95 Å) and were included in the refinement in the riding model approximation, with *U*(H) set to 1.2*U*(C). The hydroxy H-atoms were located in a difference Fourier map, and were refined with an O–H distance restraint of 0.84±0.01 Å; their temperature factors were freely refined.

### Crystal data

**Table:**

C_7_H_5_NO_4_	*Z* = 4
*M**_r_* = 167.12	*F*_000_ = 344
Triclinic, *P*1	*D*_x_ = 1.647 Mg m^−^^3^
Hall symbol: -P 1	Mo *K*α radiation λ = 0.71073 Å
*a* = 8.042 (1) Å	Cell parameters from 1123 reflections
*b* = 8.036 (1) Å	θ = 3.0–28.8º
*c* = 12.242 (2) Å	µ = 0.14 mm^−^^1^
α = 71.975 (2)º	*T* = 100 (2) K
β = 70.820 (2)º	Prism, yellow
γ = 67.323 (2)º	0.40 × 0.05 × 0.05 mm
*V* = 674.1 (2) Å^3^	

### Data collection

**Table:**

Bruker SMART APEXII diffractometer	2134 reflections with *I* > 2σ(*I*)
Radiation source: fine-focus sealed tube	*R*_int_ = 0.016
Monochromator: graphite	θ_max_ = 27.5º
*T* = 100(2) K	θ_min_ = 1.8º
φ and ω scans	*h* = −10→10
Absorption correction: none	*k* = −10→9
4245 measured reflections	*l* = −15→15
3068 independent reflections	

### Refinement

**Table:**

Refinement on *F*^2^	Secondary atom site location: difference Fourier map
Least-squares matrix: full	Hydrogen site location: inferred from neighbouring sites
*R*[*F*^2^ > 2σ(*F*^2^)] = 0.045	H atoms treated by a mixture of independent and constrained refinement
*wR*(*F*^2^) = 0.121	*w* = 1/[σ^2^(*F*_o_^2^) + (0.0598*P*)^2^ + 0.2315*P*] where *P* = (*F*_o_^2^ + 2*F*_c_^2^)/3
*S* = 0.99	(Δ/σ)_max_ = 0.001
3068 reflections	Δρ_max_ = 0.31 e Å^−^^3^
225 parameters	Δρ_min_ = −0.32 e Å^−^^3^
2 restraints	Extinction correction: none
Primary atom site location: structure-invariant direct methods	

### Fractional atomic coordinates and isotropic or equivalent isotropic displacement parameters (Å^2^)

**Table:**

	*x*	*y*	*z*	*U*_iso_*/*U*_eq_	
O1	0.4321 (2)	0.8384 (2)	0.8786 (1)	0.0221 (3)	
O2	0.9772 (2)	0.2412 (2)	1.1704 (1)	0.0225 (4)	
O3	1.1859 (2)	0.0816 (2)	1.0449 (1)	0.0224 (4)	
O4	1.1227 (2)	0.1121 (2)	0.8402 (1)	0.0204 (3)	
O5	1.0630 (2)	0.6755 (2)	0.4971 (1)	0.0220 (3)	
O6	0.4719 (2)	1.2568 (2)	0.7806 (1)	0.0277 (4)	
O7	0.2851 (2)	1.4257 (2)	0.6664 (1)	0.0231 (4)	
O8	0.3813 (2)	1.4045 (2)	0.4424 (1)	0.0199 (3)	
N1	1.0379 (2)	0.2049 (2)	1.0719 (2)	0.0173 (4)	
N2	0.4272 (2)	1.2985 (2)	0.6866 (2)	0.0189 (4)	
C1	0.5276 (3)	0.7418 (3)	0.9488 (2)	0.0175 (4)	
C2	0.6821 (3)	0.5734 (3)	0.9247 (2)	0.0157 (4)	
C3	0.7856 (3)	0.4675 (3)	1.0075 (2)	0.0160 (4)	
C4	0.9342 (3)	0.3115 (3)	0.9816 (2)	0.0153 (4)	
C5	0.9815 (3)	0.2577 (3)	0.8741 (2)	0.0162 (4)	
C6	0.8705 (3)	0.3642 (3)	0.7931 (2)	0.0193 (4)	
C7	0.7247 (3)	0.5181 (3)	0.8181 (2)	0.0185 (4)	
C8	0.9568 (3)	0.7675 (3)	0.5691 (2)	0.0181 (4)	
C9	0.8044 (3)	0.9364 (3)	0.5399 (2)	0.0157 (4)	
C10	0.6886 (3)	1.0378 (3)	0.6244 (2)	0.0157 (4)	
C11	0.5444 (3)	1.1966 (3)	0.5950 (2)	0.0157 (4)	
C12	0.5144 (3)	1.2565 (3)	0.4804 (2)	0.0155 (4)	
C13	0.6380 (3)	1.1535 (3)	0.3950 (2)	0.0174 (4)	
C14	0.7784 (3)	0.9973 (3)	0.4242 (2)	0.0173 (4)	
H4o	1.185 (4)	0.068 (4)	0.892 (2)	0.06 (1)*	
H8o	0.308 (3)	1.452 (4)	0.499 (2)	0.05 (1)*	
H1	0.5013	0.7765	1.0216	0.021*	
H3	0.7557	0.5010	1.0812	0.019*	
H6	0.8969	0.3290	0.7203	0.023*	
H7	0.6514	0.5884	0.7623	0.022*	
H8	0.9724	0.7290	0.6477	0.022*	
H10	0.7070	0.9998	0.7023	0.019*	
H13	0.6238	1.1931	0.3161	0.021*	
H14	0.8593	0.9290	0.3655	0.021*	

### Atomic displacement parameters (Å^2^)

**Table:**

	*U*^11^	*U*^22^	*U*^33^	*U*^12^	*U*^13^	*U*^23^
O1	0.0187 (8)	0.0176 (8)	0.0236 (8)	0.0041 (6)	−0.0081 (6)	−0.0052 (6)
O2	0.0246 (8)	0.0218 (8)	0.0174 (7)	−0.0016 (7)	−0.0057 (6)	−0.0057 (6)
O3	0.0171 (8)	0.0177 (8)	0.0261 (8)	0.0048 (6)	−0.0082 (6)	−0.0059 (6)
O4	0.0169 (8)	0.0172 (8)	0.0234 (8)	0.0048 (6)	−0.0073 (6)	−0.0096 (6)
O5	0.0191 (8)	0.0171 (8)	0.0251 (8)	0.0038 (6)	−0.0071 (6)	−0.0083 (6)
O6	0.0294 (9)	0.0283 (9)	0.0199 (8)	0.0037 (7)	−0.0080 (7)	−0.0121 (7)
O7	0.0175 (8)	0.0186 (8)	0.0241 (8)	0.0047 (6)	−0.0035 (6)	−0.0075 (6)
O8	0.0155 (8)	0.0171 (7)	0.0197 (8)	0.0049 (6)	−0.0051 (6)	−0.0060 (6)
N1	0.0170 (9)	0.0152 (9)	0.0187 (9)	−0.0028 (7)	−0.0058 (7)	−0.0035 (7)
N2	0.0171 (9)	0.0166 (9)	0.0189 (9)	−0.0004 (7)	−0.0029 (7)	−0.0063 (7)
C1	0.0149 (10)	0.0145 (10)	0.0201 (10)	−0.0005 (8)	−0.0023 (8)	−0.0065 (8)
C2	0.0104 (9)	0.0135 (10)	0.0198 (10)	−0.0007 (8)	−0.0025 (8)	−0.0040 (8)
C3	0.0149 (10)	0.0149 (10)	0.0163 (10)	−0.0025 (8)	−0.0033 (8)	−0.0038 (8)
C4	0.0134 (10)	0.0138 (10)	0.0167 (10)	−0.0026 (8)	−0.0055 (8)	−0.0006 (8)
C5	0.0117 (10)	0.0142 (10)	0.0204 (10)	−0.0006 (8)	−0.0038 (8)	−0.0048 (8)
C6	0.0177 (11)	0.0187 (11)	0.0197 (10)	0.0010 (8)	−0.0060 (9)	−0.0085 (8)
C7	0.0149 (10)	0.0175 (10)	0.0202 (10)	−0.0001 (8)	−0.0070 (8)	−0.0034 (8)
C8	0.0167 (10)	0.0153 (10)	0.0199 (10)	−0.0018 (8)	−0.0057 (8)	−0.0034 (8)
C9	0.0145 (10)	0.0119 (10)	0.0195 (10)	−0.0020 (8)	−0.0054 (8)	−0.0031 (8)
C10	0.0154 (10)	0.0147 (10)	0.0155 (10)	−0.0034 (8)	−0.0047 (8)	−0.0017 (8)
C11	0.0132 (10)	0.0135 (10)	0.0182 (10)	−0.0018 (8)	−0.0021 (8)	−0.0053 (8)
C12	0.0123 (10)	0.0121 (9)	0.0196 (10)	−0.0013 (8)	−0.0040 (8)	−0.0032 (8)
C13	0.0153 (10)	0.0170 (10)	0.0171 (10)	0.0000 (8)	−0.0057 (8)	−0.0043 (8)
C14	0.0150 (10)	0.0152 (10)	0.0189 (10)	0.0006 (8)	−0.0033 (8)	−0.0075 (8)

### Geometric parameters (Å, °)

**Table:**

O1—C1	1.222 (2)	C8—C9	1.475 (3)
O2—N1	1.223 (2)	C9—C10	1.380 (3)
O3—N1	1.242 (2)	C9—C14	1.406 (3)
O4—C5	1.341 (2)	C10—C11	1.397 (3)
O5—C8	1.214 (3)	C11—C12	1.405 (3)
O6—N2	1.226 (2)	C12—C13	1.408 (3)
O7—N2	1.241 (2)	C13—C14	1.368 (3)
O8—C12	1.335 (2)	O4—H4o	0.84 (1)
N1—C4	1.457 (3)	O8—H8o	0.84 (1)
N2—C11	1.447 (3)	C1—H1	0.9500
C1—C2	1.473 (3)	C3—H3	0.9500
C2—C3	1.385 (3)	C6—H6	0.9500
C2—C7	1.403 (3)	C7—H7	0.9500
C3—C4	1.394 (3)	C8—H8	0.9500
C4—C5	1.399 (3)	C10—H10	0.9500
C5—C6	1.408 (3)	C13—H13	0.9500
C6—C7	1.370 (3)	C14—H14	0.9500
			
O2—N1—O3	122.8 (2)	C10—C11—N2	117.7 (2)
O2—N1—C4	119.0 (2)	C12—C11—N2	121.0 (2)
O3—N1—C4	118.1 (2)	O8—C12—C11	126.7 (2)
O6—N2—O7	122.6 (2)	O8—C12—C13	115.7 (2)
O6—N2—C11	119.1 (2)	C11—C12—C13	117.7 (2)
O7—N2—C11	118.3 (2)	C14—C13—C12	120.9 (2)
O1—C1—C2	122.4 (2)	C13—C14—C9	120.9 (2)
C3—C2—C7	119.4 (2)	C5—O4—H4o	106 (2)
C3—C2—C1	120.4 (2)	C12—O8—H8o	110 (2)
C7—C2—C1	120.3 (2)	O1—C1—H1	118.8
C2—C3—C4	119.4 (2)	C2—C1—H1	118.8
C3—C4—C5	121.8 (2)	C2—C3—H3	120.3
C3—C4—N1	117.1 (2)	C4—C3—H3	120.3
C5—C4—N1	121.1 (2)	C7—C6—H6	119.7
O4—C5—C4	126.4 (2)	C5—C6—H6	119.7
O4—C5—C6	115.9 (2)	C6—C7—H7	119.5
C4—C5—C6	117.7 (2)	C2—C7—H7	119.5
C7—C6—C5	120.6 (2)	O5—C8—H8	118.5
C6—C7—C2	121.1 (2)	C9—C8—H8	118.5
O5—C8—C9	122.9 (2)	C9—C10—H10	120.1
C10—C9—C14	119.4 (2)	C11—C10—H10	120.1
C10—C9—C8	120.8 (2)	C14—C13—H13	119.5
C14—C9—C8	119.8 (2)	C12—C13—H13	119.5
C9—C10—C11	119.8 (2)	C13—C14—H14	119.6
C10—C11—C12	121.3 (2)	C9—C14—H14	119.6
			
O1—C1—C2—C3	−179.5 (2)	O5—C8—C9—C10	−179.1 (2)
O1—C1—C2—C7	0.0 (3)	O5—C8—C9—C14	−0.3 (3)
C7—C2—C3—C4	2.1 (3)	C14—C9—C10—C11	1.7 (3)
C1—C2—C3—C4	−178.3 (2)	C8—C9—C10—C11	−179.5 (2)
C2—C3—C4—C5	−0.4 (3)	C9—C10—C11—C12	−0.3 (3)
C2—C3—C4—N1	−179.8 (2)	C9—C10—C11—N2	−179.8 (2)
O2—N1—C4—C3	9.5 (3)	O6—N2—C11—C10	9.2 (3)
O3—N1—C4—C3	−169.8 (2)	O7—N2—C11—C10	−171.1 (2)
O2—N1—C4—C5	−170.0 (2)	O6—N2—C11—C12	−170.3 (2)
O3—N1—C4—C5	10.7 (3)	O7—N2—C11—C12	9.4 (3)
C3—C4—C5—O4	179.0 (2)	C10—C11—C12—O8	179.6 (2)
N1—C4—C5—O4	−1.6 (3)	N2—C11—C12—O8	−0.9 (3)
C3—C4—C5—C6	−1.6 (3)	C10—C11—C12—C13	−1.6 (3)
N1—C4—C5—C6	177.8 (2)	N2—C11—C12—C13	177.9 (2)
O4—C5—C6—C7	−178.7 (2)	O8—C12—C13—C14	−179.0 (2)
C4—C5—C6—C7	1.9 (3)	C11—C12—C13—C14	2.1 (3)
C5—C6—C7—C2	−0.1 (3)	C12—C13—C14—C9	−0.8 (3)
C3—C2—C7—C6	−2.0 (3)	C10—C9—C14—C13	−1.2 (3)
C1—C2—C7—C6	178.5 (2)	C8—C9—C14—C13	180.0 (2)

### Hydrogen-bond geometry (Å, °)

**Table:**

*D*—H···*A*	*D*—H	H···*A*	*D*···*A*	*D*—H···*A*
O4—H4o···O1^i^	0.84 (1)	2.13 (3)	2.676 (2)	122 (3)
O4—H4o···O3	0.84 (1)	1.91 (2)	2.638 (2)	144 (3)
O8—H8o···O5^ii^	0.84 (1)	2.10 (3)	2.687 (2)	128 (3)
O8—H8o···O7	0.84 (1)	1.94 (2)	2.635 (2)	139 (3)

Symmetry codes: (i) *x*+1, *y*−1, *z*; (ii) *x*−1, *y*+1, *z*.
